# Disseminated Leishmaniasis, a Severe Form of *Leishmania braziliensis* Infection

**DOI:** 10.3201/eid3003.230786

**Published:** 2024-03

**Authors:** Paulo R.L. Machado, Alexsandro Lago, Thiago M. Cardoso, Andréa Magalhaes, Lucas P. Carvalho, Tainã Lago, Augusto M. Carvalho, Rúbia Costa, Edgar M. Carvalho

**Affiliations:** Federal University of Bahia, Salvador, Brazil (P.R.L. Machado, A. Lago, A. Magalhaes, L.P. Carvalho, T. Lago, R. Costa, E.M. Carvalho);; National Institute of Science and Technology of Tropical Diseases (INCT-DT), Brasília, Brazil (P.R.L. Machado, L.P. Carvalho, A.M. Carvalho, E.M. Carvalho);; Fiocruz, Salvador (T.M. Cardoso, A. Magalhaes, L.P. Carvalho, A.M. Carvalho, R. Costa, E.M. Carvalho)

**Keywords:** parasites, disseminated leishmaniasis, Leishmania braziliensis, parasite dissemination, cutaneous leishmaniasis, therapy for leishmaniasis, Brazil

## Abstract

Disseminated leishmaniasis (DL) is an emergent severe disease manifesting with multiple lesions. To determine the relationship between immune response and clinical and therapeutic outcomes, we studied 101 DL and 101 cutaneous leishmaniasis (CL) cases and determined cytokines and chemokines in supernatants of mononuclear cells stimulated with leishmania antigen. Patients were treated with meglumine antimoniate (20 mg/kg) for 20 days (CL) or 30 days (DL); 19 DL patients were instead treated with amphotericin B, miltefosine, or miltefosine and meglumine antimoniate. High levels of chemokine ligand 9 were associated with more severe DL. The cure rate for meglumine antimoniate was low for both DL (44%) and CL (60%), but healing time was longer in DL (p = 0.003). The lowest cure rate (22%) was found in DL patients with >100 lesions. However, meglumine antimoniate/miltefosine treatment cured all DL patients who received it; therefore, that combination should be considered as first choice therapy.

Disseminated leishmaniasis (DL) is an aggressive form of tegumentary leishmaniasis associated with multiple and polymorphic cutaneous lesions (acneiform and inflammatory papules, nodules, and ulcers) in >2 body regions (*1*). DL has been mainly described in Brazil in patients infected with *Leishmania (Viannia) braziliensis*, but the disease is documented in other countries of South America and in the Old World. The disease may be caused by other species of *Leishmania*, including *L. mexicana amazonensis*, *L. (V) guyanensis*, *L. tropica*, and *L. major* (*2*–*5*). DL is an emerging disease and is highly endemic in the area of *L. braziliensis* transmission in northeastern Brazil. The frequency of the disease has increased >20 times in the past 30 years (*1*,*6*). When DL was initially described in this leishmaniasis-endemic area in northeastern Brazil, *L. amazonensis* was the most frequent causal agent, detected in 56.2% of the cases (*7*,*8*). However, more recently, *L. amazonensis* has not been isolated from patients in this area, and *L. braziliensis* is the only species identified in patients with American tegumentary leishmaniasis (*9*). Making distinctions between DL and diffuse cutaneous leishmaniasis (DCL) is key. Whereas DL might be caused by several *Leishmania* species, DCL is caused by *L. amazonensis* in the Americas and *L. aethiopica* in Africa. DL manifests in multiple types of lesions, such as papules, superficial nodules, and ulcerations, with few parasites in situ, whereas DCL is associated with infiltrated plaques and nodules along with a high number of parasites in the lesions (*10*).

Both parasite and host factors participate in the pathogenesis of DL. *L. braziliensis* is polymorphic, and genotypic differences in chromosomes 28 and 42 are associated with DL (*11*). Those genotypic differences among isolates of *L. braziliensis* have been associated with different clinical forms and with the severity of American tegumentary leishmaniasis and its failure to respond to meglumine antimoniate (*12*,*13*). Regarding host factors, macrophages from DL patients allow for greater parasite multiplication than cutaneous leshmaniasis (CL) cells (*14*). A parasite dissemination as observed in visceral leishmaniasis and DCL is associated with an impairment in the T-cell response (*15*,*16*). However, no clear evidence exists demonstrating that impairment in the T-cell response is the cause of parasite dissemination in DL. Approximately 20% of DL patients might experience a negative delayed-type hypersensitivity test to leishmania antigens (*17*). Although peripheral blood lymphocytes from DL patients produce fewer Th1 cytokines than those of patients with CL (*1*), immunochemistry studies of the lesions in CL and DL patients do not show differences in the cell populations and cytokine expression in those 2 forms of the disease (*17*,*18*).

Case reports of DL indicate that after a single lesion develops, dissemination occurs in >1 weeks (*1*,*8*). The number of lesions can vary widely; some patients have 10–20, and others can have >100–1,000 lesions. Nasal mucosa involvement occurs in ≈40% of DL patients (*1*,*7*,*8*). 

DL is associated with high therapeutic failure of meglumine antimoniate treatment. Studies are scarce comparing therapeutic responses to antimony in DL versus CL, as are studies investigating the efficacy of miltefosine and amphotericin B. Moreover, clinic and immunologic risk factors associated with DL are not well known. In this article, we investigated miltefosine and amphotericin B treatment of DL and CL, the phenotypic heterogeneity among DL patients when grouped by the number of lesions, and associations with distinct immunologic responses and different clinical and therapeutic outcomes.

## Materials and Methods

The study participants were 202 patients, half with DL and half with CL. All were from the leishmaniasis-endemic region of Corte de Pedra in the southeast of Bahia, Brazil. All DL patients (N = 101) whose illness was diagnosed during 2016–2020 at the Corte de Pedra Health Post were included in the study. CL patients (N = 101) were randomly assigned to the study without age or sex matching at a ratio of 1:1 DL and CL cases. The primary goal was to determine whether the number of lesions influenced the clinical outcome and response to therapy. We compared DL patients who have >50 cutaneous lesions with DL cases who have <40 lesions at the time of diagnosis.

### Case Definition and Inclusion Criteria

**Figure 1:**
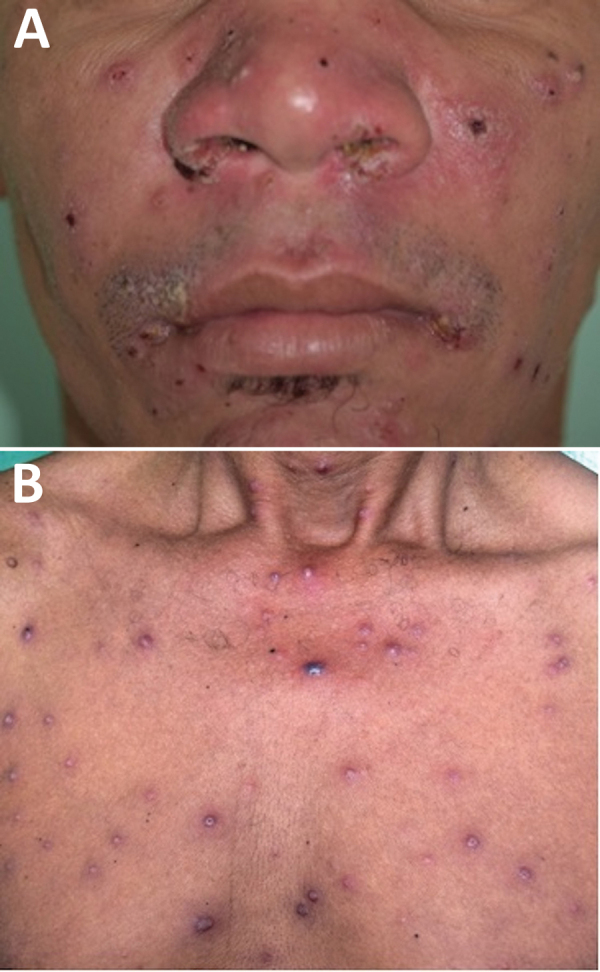
Clinical manifestation of disseminated leishmaniasis in male patient with multiple acneiform lesions, inflammatory and crusted papules in the face (A) and trunk (B), Brazil.

A DL case was defined as the presence of >10 or more cutaneous lesions over 2 or more noncontiguous body areas in a patient (*1*) (Figure 1). CL was defined by the presence of 1–3 ulcerated lesions with raised borders in any body location. The diagnosis of DL and CL was confirmed by a positive PCR result for *L. braziliensis*. We counted the cutaneous lesions and measured the diameter of the largest lesion. An ear, nose, and throat (ENT) specialist performed a nasal and pharyngeal examination to evaluate mucosal involvement.

### Skin Lesion Biopsies for Histopathology and PCR

We took skin biopsy specimens from the border of the original ulcer in both DL and CL patients. The skin fragment was obtained using a 4 mm–diameter punch after the application of a local anesthetic. The biopsy specimens were placed in formol for histopathologic studies and in RNAlater for PCR techniques. Leishmania species was determined by a serial real-time quantitative PCR system (*19*).

### *Leishmania* Antigen and Skin Test

We prepared soluble *Leishmania* antigen (SLA) as previously described (*20*). We inoculated 25 μg in 0.1 mL of SLA in the forearm and induration was determined after 48 hours. A skin test result was considered positive when the induration was >5 mm.

### Determination of Cytokines and Chemokines

We isolated peripheral blood mononuclear cells (PBMC) from heparin-treated venous blood by Ficoll-Hypaque gradient centrifugation and stimulated them with SLA as previously described (*21*). In brief, after washing 3 times in 0.9% NaCl, we resuspended cells in RPMI 1640 Medium (ThermoFisher Scientific) supplemented with 10% fetal bovine serum, 100 IU/mL penicillin, and 100 μg/mL streptomycin. Cells were adjusted to 3 × 10^6^ cells/mL, put in 24-well plates, and stimulated with SLA (5 μg/mL). After incubation for 72 hours at 37°C and 5% CO_2_, we collected and stored supernatants at –20°C. The levels of interferon (IFN) γ, tumor necrosis factor (TNF), interleukin (IL) 1β, IL-10, chemokine ligand (CXCL) 9, and CXCL-10 were measured by the ELISA sandwich method with reagents from BD Bioscience and the results were expressed as picograms per milliliter (*22*).

### Treatment and Cure Criteria

As recommended by the Brazil Ministry of Health, the standard therapy was meglumine antimoniate (20 mg/kg) for 30 days for DL and 20 days for CL. However, DL is common in patients >50 years of age, and those patients should be treated with amphotericin B or miltefosine to reduce adverse reactions. Of the 202 study participants, 82 DL and all 101 CL patients were treated with meglumine antimoniate. We evaluated patients every 30 days until cure. We registered the number and size of lesions and noted appearance of new lesions, occurrence of mucosal disease, and adverse reactions at each visit. We defined cure as complete epithelization of all lesions without infiltrated borders 90 days after initiating therapy.

Age of >50 years, heart disease, and kidney failure are contraindications for the use of meglumine antimoniate. In this study, 19 DL patients did not receive meglumine antimoniate and were treated with available alternative drugs: 3 patients received deoxycholate amphotericin B (20–30 mg/kg weight; 6 patients received liposomal amphotericin B (35–40 mg/kg weight; 5 patients received miltefosine (2.5 mg/kg/d [maximum dose 150 mg/d] for 28 days); and 5 patients received miltefosine (same dosing) combined with meglumine antimoniate (20 mg/kg weight for 30 d). Patients who failed to respond to meglumine antimoniate received a second course of the same dose. Those who failed to respond to miltefosine or amphotericin B received liposomal amphotericin B (35 mg/kg weight).

### Ethical Considerations

This study was approved by the Institutional Review Board of the Federal University of Bahia (document of approval CAAE 62974916.8.0000.5577). Written consent was obtained from all participants.

## Results

### Clinical Profile of DL and CL Patients

DL patients were older than CL patients; men predominated in both groups, but the percentage of men was substantially higher in the DL group (Table 1). The duration of disease before diagnosis was longer in patients with DL. Both the frequency of patients with positive leishmania skin test (p = 0.0001) and the induration size (p = 0.0001) were higher for CL than for DL. After 1 course of meglumine antimoniate therapy, 44% of DL patients were cured, compared with 60% of CL patients. The healing time was significantly shorter for CL than for DL (110.8 + 7.7 vs. 177 + 19.6 days; p = 0.001). Mucosal disease associated with cutaneous lesions was observed in 33 (40.7%) of 81 DL patients, as determined by an ENT specialist. Those lesions were characterized as nodular or superficial ulcers in the nasal mucosa.

**Table 1:** Demographic, clinical, laboratory, and therapeutic characteristics of DL and CL patients in study of leishmaniasis immune response and clinical and therapeutic outcomes, Corte de Pedra Health Post, Brazil, 2016–2019*

Characteristic	DL, n = 101	CL, n = 101	p value
Age, y	39.5 + 14.8	32 + 13.3	0.0002†
Sex, no. (%) patients.			
M	88 (87)	69 (68)	0.04‡
F	13 (13)	23 (32)	0.04‡
Duration of disease until diagnosis, d	52.7 + 2.7	41 + 1.7	0.0003†
No. lesions	113.6 + 210	1.4 + 0.7	<0.0001†
Biggest lesion size, mm^2^	775.6 + 2,190	392.7 + 283.4	NS
Lymphadenopathy, no. positive/no. tested (%)	47/93 (50.5)	61/101 (60.4)	NS
LST size, mm^2^	102.3 + 96.5	213.6 + 126.9	0.0001†
LST , no. positive/no. tested (%)	64/97 (66)	101/101 (100)	0.0001‡
PCR. no. positive/no. tested (%)	84/91 (92)	101/101 (100)	NS
Cure rate, no. cured/no. treated (%)§	34/78 (44)¶	44/101 (60)	NS
Healing time, d§	177 + 19.6	110.8 + 7.7	0.001†

*Values are mean + SD unless otherwise indicated. CL, cutaneous leishmaniasis; DL, disseminated leishmaniasis; LST, *Leishmania* skin test, NS, not significant. †By unpaired t-test. ‡By Fisher exact test. §After 1 standard course of meglumine antimoniate (20 mg/kg/d) for 20 d (CL) or 30 d (DL). ¶Six patients had no outcome data, irregular use, or discontinuation.

### Cytokine and Chemokine Profile in DL

**Figure 2:**
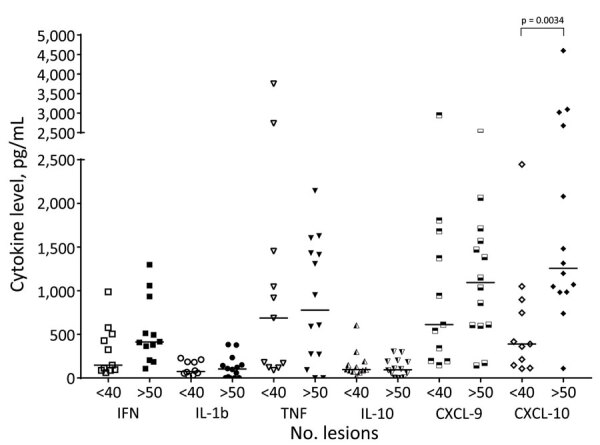
Systemic production of chemokines and cytokines among disseminated leishmaniasis (DL) patients with >50 and <40 lesions, Corte de Pedra Health Post, Brazil, 2016–2019. Peripheral blood mononuclear cells from 11 patients with <40 lesions and 14 patients with >50 lesions were cultured in the presence of soluble *Leishmania* antigen (5 µg/mL) for 72 hours. Cytokine levels in culture supernatants were measured by ELISA. Horizontal lines represent median values. CXCL, chemokine ligand; IFN, interferon; IL, interleukin; TNF, tumor necrosis factor.

We have previously shown that DL patients produce lower levels of IFN-γ and TNF in supernatants of PBMC stimulated with SLA than do CL patients (*21*). To better understand the pathogenesis of DL and to determine whether the number of lesions in DL was associated with cytokine production, we measured IFN-γ, TNF, IL-1β, IL-10, CXCL-9, and CXCL-10 in supernatants of PBMC cultures stimulated with SLA in DL patients who had <40 lesions (DL<40) and in those with >50 lesions (DL>50) (Figure 2). No difference was noted regarding the production of IFN-γ, TNF, IL-1β, IL-10, and CXCL-9 between the 2 groups, but CXCL-10 was higher (p = 0.0034) in supernatants of lymphocyte cultures of DL>50 patients (1,742 + 1,206 pg/mL) than in DL<40 patients (626 + 684.4 pg/mL).

### Demographic and Clinical Features of DL>50 Patients and DL<40 Patients

During the study period, we diagnosed DL>50 in 40 patients and DL<40 in 55 patients (Table 2). DL>50 was associated with older age and shorter duration of illness. The time between the appearance of the first lesion and dissemination was similar in the 2 groups. Systemic symptoms such as fever, chills, and headache were present in most cases (76% of DL>50 cases and 70% of DL<40). Although not a significant difference, the frequency of mucosal disease was higher in DL>50 patients (44%) than in DL<40 patients (31%). Cure rate was 30% in DL>50 patients and 56% in DL<40 patients after a single course of meglumine antimoniate (p = 0.03). Moreover, the healing time in DL>50 patients was longer (p = 0.001) than in DL<40 patients. 

**Table 2:** Demographic, clinical, laboratory, and therapeutic aspects of DL patients according to number of lesions in study of leishmaniasis immune response and clinical and therapeutic outcomes, Corte de Pedra Health Post, Brazil, 2016–2019*

Characteristic	DL with >50 lesions, n = 40	DL with <40 lesions, n = 55	p value	
Age, y	44.5 + 13.3	35.3 + 14.2	0.0018†	
Sex				
M	34 (85)	49 (89)	NS	
F	6 (15)	6 (11)	NS	
Duration of disease, d	46 + 3.4	58 + 4.0	0.031†	
Dissemination time, d	21 + 2.3	26 + 4.4	0.40†	
Systemic symptoms, no. positive/no. tested (%)	29/38 (76)	28/40 (70)	NS	
No. lesions	252 + 45.1	22 + 1.0	<0.0001†	
Largest lesion area, mm^2^	1181 + 581.2	905 + 415.3	0.69#	
Lymphadenopathy, no. positive/no. tested (%)	13/26 (50)	25/51 (49)	NS	
Mucosal involvement, no. positive/no. tested (%)	16/36 (44.4)	12/39 (31)	NS	
LST area, mm^2^	156 + 84.7	141 + 71.4	0.47†	
LST, no. positive/no. tested (%)	24/39 (62)	36/54 (67)	NS	
PCR, no. positive/no. tested (%)	24/26 (92)	44/49 (90)	NS	
Cure rate, no. cured/no. treated (%)‡	7/21 (33)	23/41 (56)	0.03§	
Healing time, d	218 + 203	109 + 95	0.0018†

*Values are no. (%) or mean + SD unless otherwise indicated. DL, disseminated leishmaniasis; LST, *Leishmania* skin test, NS, not significant. †By unpaired t-test. ‡After 1 standard course of meglumine antimoniate (20 mg/kg/d) for 30 d. §By Fisher exact test.

**Figure 3:**
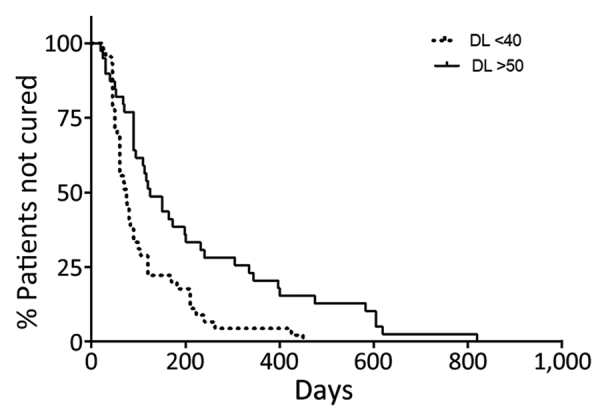
Kaplan-Meyer curve showing time to cure in the 2 groups of DL patients treated with meglumine antimoniate in monotherapy, Corte de Pedra Health Post, Brazil, 2016–2019. Patients with >50 lesions (n = 40) and <40 lesions (n = 55) were treated with meglumine antimoniate (20 mg/kg/d) for 20 days (p = 0.0012 by log-rank test). DL, disseminated leishmaniasis.

Because the classification of the 2 patient groups was arbitrary, we performed other comparisons to better evaluate the effect of the number of lesions in therapeutic response to meglumine antimoniate. The cure rate in persons with DL who had <20 lesions was 65% and for DL patients with >100 lesions was 22% (p = 0.003). The cure rate in patients with DL<40 (56%) was higher than in patients with DL with >100 lesions (22%) (p = 0.006). The cure rate progressively decreased according to the number of lesions; the cure rate was 65% in patients with <20 lesions, 56% in patients with <40 lesions, 30% in patients with >50 lesions, and 22% in persons with >100 lesions. The Kaplan-Meyer curve (Figure 3) shows that DL<40 patients healed in less time than did DL>50 patients.

### Therapeutic Response of DL to Amphotericin B and Miltefosine

We demonstrate the clinical features, cure rate at day 90, and healing time of patients who were treated with amphotericin B, miltefosine, or miltefosine combined with meglumine antimoniate and in those who only received meglumine antimoniate (Table 3). The demographic and clinical features were similar in the 4 groups of patients; the number of lesions was lower in patients treated with miltefosine alone. The healing time was shorter (p<0.01) for persons who received meglumine antimoniate plus miltefosine than for patients in the other groups. Moreover, all patients who received the combined therapy were cured before day 90, and 4 (80%) of them were cured on day 60.

**Table 3:** Clinical profile and response to therapy of disseminated leishmaniasis patients treated with amphotericin b, miltefosine, and miltefosine plus meglumine antimoniate in study of leishmaniasis immune response and clinical and therapeutic outcomes, Corte de Pedra Health Post, Brazil, 2016–2019*

Treatment†	Age, y	% Men	Illness duration, d	No. lesions	Cure rate on day 90	Healing time
Amphotericin B, n = 9	59 + 5.1	88.8%	54 + 16.9	350 + 489.1	55.5%	137 + 1113
Miltefosine, n = 5	54 + 9.2	100%	54 + 9.2	49 + 18.8	40%	96 + 27.9
Miltefosine + MA, n = 5	57 + 9.3	80%	42 + 7.5	181 + 204.2	100%	53 + 18.3
MA, n = 78	39 + 16.7	84.6%	53 + 6.1	116 + 217.3	44%	177 + 19.6
p value	0.51‡	0.47§	0.32¶	0.26¶	NA	0.01¶

*Values are mean + SD unless otherwise indicated. MA, meglumine antimoniate; NA, not applicable. †The total number of disseminated leishmaniasis cases with therapeutic outcome was 97. We have no follow-up data for 4 patients. ‡By student t-test. §Fisher exact test. ¶Kruskal-Wallis test.

## Discussion

DL is a severe disease caused by *L. braziliensis* that is characterized by a large number of cutaneous lesions, occurrence of both skin and nasal mucosal disease, and high rate of therapeutic failure to meglumine antimoniate, the drug that is recommended to treat leishmaniasis in Latin America (*17*). The pathogenesis of DL is not completely understood; clinical findings and response to therapy is based on case series consisting of small numbers of patients (*7*,*8*). We compared clinical features and response to therapy in 101 CL patients and 101 DL patients and evaluated the association between number of lesions with clinical findings, cytokine production, and outcome of therapy. We confirmed that DL patients are predominantly male, that DL is highly associated with mucosal disease, and that treatment with meglumine antimoniate has a high rate of failure. The number of lesions in DL cases was variable; increased numbers of lesions were associated with age, duration of illness, long healing time, and production of CXCL-10 in PBMC supernatants stimulated with SLA. Moreover, in a small number of patients, we observed that combined therapy with miltefosine and meglumine antimoniate resulted in a higher cure rate of DL than other forms of therapy.

In this study, DL patients were older than CL patients, but we also identified a large number of DL case-patients <50 years of age and many women with DL, which differed from previous reports (*7*,*14*). The cases of DL in our leishmaniasis-endemic area have spread from inner regions to other parts, suggesting parasites that cause DL are spreading and that transmission is occurring in peridomicile areas rather than only in farms, as previously described (*11*,*23*). Those changes in epidemiology might have influenced the increasing occurrence of DL in young patients and in women. The low cure rate of CL with meglumine antimoniate is a major public health problem in our area; the failure rate has increased from 10% to >50% in the past 40 years (*24*–*27*). In this study, the cure rate by meglumine antimoniate was similar in CL and DL cases, but the healing time was longer for DL patients than for CL patients.

The immune response at the lesion site and histopathologic features are similar in DL and CL, but frequency of positive *Leishmania* skin test was lower in DL than in CL (*17*,*18*,*28*). In addition to the less frequent positive skin tests, the size of the skin test reaction was smaller in DL than in CL. The contrast between the similarity of the immune response at the lesion site in DL and CL and the poor Th1 immune response observed in DL in vivo and in vitro tests to evaluate T-cell response argue against an impairment in the Th1 immune response (*17*). Because of migration of most antigen-reactive cells to the multiple infected skin lesions, it is likely those cells are lacking in peripheral blood and in the other tissues, decreasing T-cell responses in the delayed-type hypersensitivity test and in blood cells.

Regarding the histopathology and cytokine production, DL lesions have fewer granuloma and higher frequencies of B cells and plasma cells than CL ulcers (*8*,*29*). More recently, we have shown that SLA IgG and IgG2 titers are higher in DL than in CL (*30*). Moreover, we demonstrated a correlation between number of lesions and *L. braziliensis* IgG2 production in DL patients (*29*). In this study, most cytokine levels were similar in the supernatants of PBMC stimulated with SLA from DL and CL, as well in supernatants of cells from DL patients with >50 lesions or <40 lesions, but CXCL-10 levels were higher in DL patients with >50 lesions. The inflammatory response is exaggerated in DL patients (*14*). CXCL-10 is expressed in blood cells, and its receptor, chemokine receptor 3, is expressed in tissues. The interaction of those chemokines enables macrophages and T cells to pass to the lesion site, increasing the inflammatory response (*30*,*31*), which suggests that CXCL-10 might contribute to the inflammatory response in DL patients and to parasite dissemination.

The high number of cutaneous lesions and the concomitant occurrence of cutaneous and mucosal involvement is a hallmark of DL. We compared the clinical features and cure rate in DL patients who had <40 lesions with patients who had >50 lesions. We left a gap between 40 and 50 lesions because very small lesions might be missed on routine clinical examination. Patients with >50 lesions were older and had shorter duration of illness, but we found no difference between the 2 groups of patients regarding symptoms associated with systemic manifestations. The frequency of mucosal leishmaniasis was similar in those with >50 and <40 lesions, indicating that the number of lesions is not a biomarker of mucosal disease in DL patients. Mucosal leishmaniasis is one of the more severe forms of *L. braziliensis* infection, characterized by ulcerated lesions, rupture of the nasal septum, and destruction of the facial structure (*32*). Mucosal leishmaniasis usually occurs weeks or even years after a cutaneous ulcer, but in a recent large series of patients with mucosal leishmaniasis, we found that 30% of cases had concomitant cutaneous and mucosal disease (*33*,*34*). The severity of mucosal disease in *L. braziliensis* infection has been classified by stages ranging from 1 to 5 (*35*). A nodule is the first sign of mucosal involvement, followed by superficial and deep ulcer cutaneous, nasal septum perforation, and destruction of the facial structure. In DL, patients’ mucosal disease is characterized by nodules and superficial ulcers; the mild mucosal disease and the initiation of therapy before nasal tissue is destroyed might contribute to the curing of mucosal lesions in <60 days for most DL patients.

The cure rate in patients who had >50 lesions was significantly lower than for persons with <40 lesions; only 30% of patients with >50 lesions were cured with 90 days of therapy. Moreover, a higher number of lesions was associated with prolonged healing time. Most DL patients were treated with meglumine antimoniate, but a limited number of patients were treated with amphotericin B, miltefosine, or miltefosine combined with meglumine antimoniate. We have previously shown that miltefosine is more effective than meglumine antimoniate in CL patients (*27*,*36*). However, monotherapy with miltefosine only cured 40% of DL patients. All 5 patients who used miltefosine plus meglumine antimoniate were cured, and healing time was short. Amphotericin B is known to be the best drug for therapy in American tegumentary leishmaniasis, and liposomal amphotericin B in a total dose ranging from 17 to 37 mg/kg cured 70% of DL patients by day 90 (*37*). In this study, only 4 of 9 patients treated with this drug did not achieve cure by day 90, although all were eventually cured without the use of other drugs. Patients taking amphotericin B who did not achieve cure by day 90 had more severe disease; in 3 of those patients, the number of lesions ranged from 405 to 1,500.

The limitations of this study are that not all patients had an ENT examination, follow-up care was not completed in ≈8% of DL patients treated with meglumine antimoniate, and alternative therapies were only used in a limited number of patients. Moreover, treatment with amphotericin B is very difficult in this leishmaniasis-endemic area, and the effective dose of this drug was only achieved 60–90 days after initiating therapy. However, this study followed a much larger number of DL patients prospectively than previous studies, and new information was obtained. Most DL patients were <40 years of age, and despite mucosal disease occurring in a high frequency, the mucosal lesions were mild and responded well to therapy. Despite an increase in failure of meglumine antimoniate therapy observed in CL patients in this area, healing time was longer for DL patients than for CL patients, and the number of lesions in DL patients was associated with increased treatment failure. In addition, we extend previous observations regarding the therapeutic response in DL. The high rate of therapeutic failure and the long healing time of DL patients treated with meglumine antimoniate indicates that alternative drugs or polychemotherapy should be used for the treatment of DL. Although further testing in a large number of DL patients is needed, our preliminary observation of a high cure rate in patients who received meglumine antimoniate combined with miltefosine supports use of those drugs as first choice therapy.
